# Effects of noise exposure on young adults with normal audiograms II: Behavioral measures

**DOI:** 10.1016/j.heares.2017.10.007

**Published:** 2017-12

**Authors:** Garreth Prendergast, Rebecca E. Millman, Hannah Guest, Kevin J. Munro, Karolina Kluk, Rebecca S. Dewey, Deborah A. Hall, Michael G. Heinz, Christopher J. Plack

**Affiliations:** a Manchester Centre for Audiology and Deafness, University of Manchester, Manchester Academic Health Science Centre, M13 9PL, UK; b NIHR Manchester Biomedical Research Centre, Central Manchester University Hospitals NHS Foundation Trust, Manchester Academic Health Science Centre, Manchester, M13 9WL, UK; c Sir Peter Mansfield Imaging Centre, School of Physics and Astronomy, University of Nottingham Nottingham, NG7 2RD, UK; d National Institute for Health Research (NIHR) Nottingham Biomedical Research Centre, Nottingham, NG1 5DU, UK; e Otology and Hearing Group, Division of Clinical Neuroscience, School of Medicine, University of Nottingham, Nottingham, NG7 2UH, UK; f Department of Speech, Language, & Hearing Sciences and Biomedical Engineering, Purdue University, West Lafayette, IN, 47907, USA; g Department of Psychology, Lancaster University, Lancaster, LA1 4YF, UK

**Keywords:** Cochlear synaptopathy, Hidden hearing loss, Noise-induced hearing loss, Speech-in-noise, Psychophysics

## Abstract

An estimate of lifetime noise exposure was used as the primary predictor of performance on a range of behavioral tasks: frequency and intensity difference limens, amplitude modulation detection, interaural phase discrimination, the digit triplet speech test, the co-ordinate response speech measure, an auditory localization task, a musical consonance task and a subjective report of hearing ability. One hundred and thirty-eight participants (81 females) aged 18–36 years were tested, with a wide range of self-reported noise exposure. All had normal pure-tone audiograms up to 8 kHz. It was predicted that increased lifetime noise exposure, which we assume to be concordant with noise-induced cochlear synaptopathy, would elevate behavioral thresholds, in particular for stimuli with high levels in a high spectral region. However, the results showed little effect of noise exposure on performance. There were a number of weak relations with noise exposure across the test battery, although many of these were in the opposite direction to the predictions, and none were statistically significant after correction for multiple comparisons. There were also no strong correlations between electrophysiological measures of synaptopathy published previously and the behavioral measures reported here. Consistent with our previous electrophysiological results, the present results provide no evidence that noise exposure is related to significant perceptual deficits in young listeners with normal audiometric hearing. It is possible that the effects of noise-induced cochlear synaptopathy are only measurable in humans with extreme noise exposures, and that these effects always co-occur with a loss of audiometric sensitivity.

## Highlights

• Large study on the effects of lifetime noise exposure in normal-hearing young adults.
• Performance on a range of behavioral tasks unrelated to noise exposure history.
• Effects of cochlear synaptopathy not evident in young audiometrically normal cohort.

## 1 Introduction

Cochlear synaptopathy due to noise exposure (often referred to as “hidden hearing loss”) was demonstrated in a mouse model by Kujawa and Liberman (2009). In the base of the cochlea, 50% of synapses were lost between inner hair cells (IHCs) and auditory nerve (AN) fibers after a 2-h exposure to 100 dB SPL noise (8–16 kHz). Post-exposure, measures of absolute auditory sensitivity were unaffected but a permanent decrease in the amplitude of wave I of the auditory brainstem response (ABR), reflecting decreased auditory nerve activity, was seen in response to moderate- and high-intensity stimuli. The synaptic loss was subsequently found to preferentially affect the high-threshold, low spontaneous-rate (SR) AN fibers (Furman et al., 2013).

A loss of cochlear synapses due to noise exposure, in the presence of almost unaffected threshold sensitivity, has also been demonstrated in a range of other rodents (e.g. guinea pig, Lin et al., 2011; chinchilla, Hickox et al., 2015, Hickox et al., 2017; rat, Möhrle et al., 2016). However, pure synaptopathy may be more difficult to produce in primates. In the macaque model, high noise exposures (108 dB SPL or greater) may be required for around 4 h to produce supra-threshold reductions in the amplitude of wave I of the ABR (Valero et al., 2017). A number of review articles give a thorough account of the progression from the initial seminal work in the mouse, to the current understanding in the field (Kobel et al., 2017, Liberman and Kujawa, 2017, Plack et al., 2014, Plack et al., 2016). However, the initial account of cochlear synaptopathy as described in the mouse model may not be translated into an analogous human pathology in a straightforward way (Hickox et al., 2017).

The evidence for noise-induced cochlear synaptopathy in human listeners is somewhat sparse and inconsistent. Stamper and Johnson (2015a) first provided evidence for reductions in ABR wave I amplitude with greater noise exposure in audiometrically normal human listeners. However, audiograms were only measured up to 8 kHz, and it is possible that high-frequency hair cell loss affected wave I amplitudes in the more exposed listeners (Don and Eggermont, 1978). Furthermore, there was a confound of sex in that the most noise exposed listeners in the cohort were male, and males also tend to show smaller ABR amplitudes due to factors such as skull thickness and head size (Picton et al., 1981, Jerger and Hall, 1980). In a subsequent letter, Stamper and Johnson (2015b) analyzed their data for the highest click level (90 dB nHL) for the two sexes independently. The relation between wave I amplitude and a 12-month noise exposure estimate persisted for females, but not males.

Recently, Bramhall et al. (2017) reported that non-veteran firearm users and veterans with high levels of noise exposure have reduced wave I amplitudes relative to lower noise exposed veterans and non-veterans without a history of firearm use. All groups had similar otoacoustic emissions and normal audiograms up to 8 kHz, although noise-exposed veterans showed an average elevation of audiometric threshold (averaged across 2000, 3000 and 4000 Hz) of 7.3 dB compared to non-veterans. High-frequency audiometric testing (>8 kHz) was only performed on 59% of participants, and so the contribution of high-frequency hearing loss is uncertain.

In contrast to these findings of lower wave I amplitudes with greater noise exposure, we conducted a large-scale study (N = 126) of young, normal-hearing adults and found no significant relation between lifetime noise exposure and ABR wave I amplitude for either males or females (Prendergast et al., 2017). These findings were replicated in a subsequent study from the same laboratory (Guest et al., 2017). Such negative findings are concordant with Liberman et al. (2016), who reported no significant difference in wave I amplitude between their high- and low-exposure groups. However, they did find a difference in the ratio of the summating potential (SP) relative to the action potential (AP; effectively wave I). A larger SP/AP ratio was found for the high-noise group, due mainly to a higher SP for that group. Since the SP is thought to be generated by the hair cells (Kiang and Peake, 1960), a high SP/AP ratio is consistent with synaptopathy. However, it remains unclear how a loss of cochlear synapses would lead to enhancement of the SP, or how the SP would be affected by the substantial high-frequency audiometric deficit observed in the high-noise group.

Although the electrophysiological results in humans are mixed, with a number of studies showing discordant findings, it is possible that ABR measures are relatively insensitive to cochlear synaptopathy (Bourien et al., 2014, Prendergast et al., 2017) and that behavioral measures of auditory coding are more sensitive. Furthermore, important questions remain regarding the behavioral consequences of cochlear synaptopathy, and, more generally, regarding whether or not noise exposure is related to behavioral deficits in humans in the presence of normal audiometric thresholds.

There is existing evidence that noise exposure leads to impaired performance on a range of behavioral auditory tasks for listeners with normal audiograms, although some of these studies are confounded by age and audiometric differences between the groups. Alvord (1983) measured identification scores for words in background noise presented at 60 dB HL. Noise-exposed listeners performed on average 10% more poorly than non-noise-exposed listeners but the groups were not sex-, age- or audiogram-matched. Kujala et al. (2004) used a task of deviant syllable detection and for age- and audiogram-matched groups, found a decrease in performance for noise-exposed listeners. Kumar et al. (2012) compared noise-exposed train drivers with age-matched controls and found that the noise-exposed group had deficits in amplitude modulation detection and speech recognition in background babble using stimuli presented at 80 dB SPL. However, it is not clear from the paper if the groups were audiometrically matched and thus the observed differences may be explained by a more standard, and measurable, loss of audiometric sensitivity. Hope et al. (2013) compared noise-exposed Air Force pilots with non-exposed Air Force administrators and found a deficit in speech-in-noise (vowel-consonant-vowel stimuli) perception thresholds for the noise-exposed group. There was no difference between the groups on other auditory tasks including simultaneous masking, backward masking and frequency discrimination. Though the groups were audiometrically matched to within 2.2 dB, this only included frequencies up to and including 4000 Hz.

Stone et al. (2008) used a task in which normal-hearing listeners were required to discriminate envelopes with different noise statistics at low sensation levels. Noise-exposed listeners showed a deficit in performance compared to non-noise-exposed controls, though these differences were observed at low sensation levels and therefore would not be dependent primarily on low-SR fibers. This evidence is therefore difficult to reconcile with the animal model of noise-induced synaptopathy. Liberman et al. (2016) demonstrated performance deficits on a speech-in-noise task for noise-exposed listeners relative to less exposed controls. However, stimuli were presented at 35 dB HL, again suggesting minimal contributions of low-SR fibers to performance as at this sound intensity the high-SR fibers are unlikely to be saturated and thus efficient coding is not primarily dependent on low-SR fibers. Le Prell and Lobarinas (2016) examined measures of recreational noise exposure for groups of audiometrically normal young people, divided based on performance on a measure of word recognition in noise. The groups did not differ significantly in preferred listening level, nor in number of sources of high-level noise they were exposed to. Additionally, no reliable relation was observed between perceptual performance and the reported incidence of temporary threshold shift. Finally, Yeend et al. (2017) reported results from a cohort of 30–60 year-old listeners. The primary aim was to characterize the perceptual deficits associated with increased noise exposure. The authors reported no link between lifetime noise exposure and performance on any of the psychophysical or speech tasks. High-frequency hearing thresholds were predictive of speech-in-noise performance.

In this article we describe a series of behavioral measures that we collected concurrent with the electrophysiological data presented in Prendergast et al. (2017). We consider whether an estimate of lifetime noise exposure is able to predict performance on a range of behavioral tasks for young listeners with normal audiograms. By doing so we hoped to determine which, if any, behavioral tasks may be affected by synaptopathy, based on the assumption that greater lifetime noise exposure is a proxy for increased cochlear synaptopathy. As well as psychophysical tasks used to examine the coding fidelity of a listener's auditory system, we included tasks more representative of real-world listening ability, including speech-in-noise tasks, an auditory localization task, and a musical consonance task. Finally, we included the Speech, Spatial and Qualities of Hearing Scale (SSQ; Gatehouse and Noble, 2004) questionnaire to measure self-reported listening ability. Listeners with normal audiograms often report that they have listening difficulties (e.g. Davis, 1989), and it may be important to capture aspects of more general listening ability, beyond specific laboratory tasks.

The rationales for the tasks and stimuli chosen are based in part on what is known about noise-induced synaptopathy in the animal model. A compelling overview of how this may express itself in humans is provided by Bharadwaj et al. (2014), in which the authors predict that a loss of low-SR fibers will lead to a reduction in temporal coding, with poorer representations of acoustic signals in the auditory nerve. This would then lead to a reduction in the ability to discriminate subtle timing differences, for example in a frequency discrimination or inter-aural phase discrimination task. Bharadwaj et al. (2015) demonstrated that subcortical EEG measures, the ability of a listener to detect differences in the phase of a stimulus between ears, and amplitude modulation detection performance all co-vary, seemingly due to individual differences in temporal coding. Although the study contained a crude measure of noise exposure history, which suggested that noise-exposed participants have weaker evoked responses and elevated behavioral thresholds, the authors were cautious in concluding that noise-induced synaptopathy was the primary factor. In this study we included comparable tasks to ascertain if temporal coding varies as a function of lifetime noise exposure.

The effects of noise exposure were predicted to be most readily observed in response to high-level stimuli, as these would lead to saturation of high-SR fibers and therefore any differences in residual coding would be carried by the population of low-SR fibers. This approach is based on the low-SR hypothesis, supported by data from Furman et al. (2013), and assumes that this fiber group is critically important to the encoding of high-intensity sounds. Hence, for most tasks we used both low- and high-level conditions in order to provide a differential measure of the effects of synaptopathy. Note that this approach is insensitive to synaptopathy if high-SR fibers are able to encode high-level sounds by modulating their firing patterns (Young and Sachs, 1979). In addition, noise-induced audiometric hearing loss, caused mainly by damage to the outer hair cells (OHCs), typically manifests in the 3000–6000 Hz region (Toynbee, 1860, McBride and Williams, 2001). Therefore, for a number of tasks, stimuli with frequency components in two spectral regions were used, with the assumption that synaptopathy is most likely to occur in the same frequency range as noise-induced outer hair cell dysfunction. Hence, for the psychophysical tasks, we measured performance at 255 Hz and 4000 Hz to provide another differential measure. Differential measures may help to control for the effects of variability between individuals due to factors unrelated to synaptopathy (Plack et al., 2016).

Finally, musical training is related to enhanced performance on some auditory tasks (Parbery-Clark et al., 2009, Zendel and Alain, 2009). Yeend et al. (2017) reported that sensitivity to temporal fine structure and amplitude modulation was enhanced in musically trained listeners. In order to control for the effects of musical experience in our cohort, we included an estimate of the number of years during which a musical instrument was played regularly.

## 2 Methods

### 2.1 Participants

One hundred and thirty-eight participants (82 females), with a wide range of noise exposures, were tested, 123 of whom were also tested as part of an electrophysiological study of noise-induced synaptopathy (Prendergast et al., 2017). Participants were recruited mainly via a publicly available University of Manchester website listing active research projects. Advertisements were also placed in a number of bars and music venues in Manchester city center. All participants exhibited clinically normal audiometric thresholds (see section 2.3). Males had a mean age of 23.3 years (range, 18–36) and females had a mean age of 23.1 years (range, 18–36). The procedures were approved by the University of Manchester Research Ethics Committee and all participants gave informed consent (project number 14163).

### 2.2 Noise exposure

Lifetime noise exposure was estimated using a structured interview developed to assess the effectiveness of the UK noise at work regulations (Lutman et al., 2008). The specific implementation used is described fully by Prendergast et al. (2017). In summary, participants are asked to consider any high-noise (above ∼ 85 dBA) environments/activities to which they have exposed themselves with a degree of repeatability over the course of their lifetime. The duration, frequency and level of exposure is estimated from discussion with the participant (including any attenuation from hearing protection used) and entered into the following formula:

*U* = 10^(L−*A*−90)/10^ x *Y* x *W* x *D* x *H* / 2080,

where *U* is cumulative noise exposure, *L* is estimated noise exposure level in dBA, *A* is attenuation provided by hearing protection in dB, *Y* is years of exposure, *W* is weeks of exposure per year, *D* is days of exposure per week, *H* is hours of exposure per day, and 2080 corresponds to the number of hours in a working year. One noise exposure unit is equivalent to exposure for 1 year to a working daily level of 90 dBA. For our purposes, we used the raw noise immission units and these were log transformed to produce a normal distribution. Each such logarithmic unit is a factor of 10 in terms of lifetime exposure energy.

### 2.3 Pure tone audiometry

Pure tone audiometry was performed for each ear separately at octave frequencies between 0.25 and 8 kHz in accordance with the British Society of Audiology (2011) recommended procedure. Thresholds were measured using VIASYS GSI-Arrow audiometers coupled to TDH-39P supra-aural headphones, with MX41 cushions. The audiometric criterion for inclusion in the study was audiometric thresholds <25 dB HL in both ears at all test frequencies.

High-frequency audiometry was also performed at 16 kHz using a Creative E-MU 0202 USB soundcard. Sounds were played over Sennheiser HDA 200 circum-aural headphones designed for high-frequency audiometry. The sound stimulus was a quarter-octave wide band of noise centered at 16 kHz and converted from digital to analog at a sample rate of 48 kHz using a 24-bit depth. Stimuli were 220 ms in duration (including 10-ms raised-cosine ramps) and there was an inter-stimulus interval of 500 ms. A three-alternative forced-choice procedure was used, with a two-down, one-up staircase adaptively setting the stimulus level. Stimulus level was varied arithmetically using a step size of 4 dB for the first four reversals and 2 dB for the following 10 reversals. Thresholds were calculated by averaging the levels of the final 10 reversals from a single run.

Participants were asked if they suffered from tinnitus. If a positive response was given, participants were asked further questions to determine if this constituted prolonged tinnitus and when it was last perceived. If participants reported this percept regularly (at least every month), they were recorded as having tinnitus.

### 2.4 Behavioral tasks

All stimuli were presented using a Creative E-MU 0202 USB soundcard and Sennheiser HD650 circum-aural headphones. All stimuli were presented diotically, except for the interaural phase difference (IPD) task and the localization task. Many of the behavioral tasks were performed in both a low- and high-frequency region (255 Hz and 4000 Hz respectively, denoted “L” and “H”) and also at a low and high sound intensity (40 and 80 dB SPL, denoted “40” and “80”). This was done to test the specific hypothesis that high-threshold, high-frequency fibers are preferentially affected by lifetime noise exposure. Unless specified, a two-down, one-up adaptive track was used, and the first four reversals were made using one step size and the final 10 using a smaller step size. Thresholds were calculated from the average of the tested parameter values at the final 10 reversals. Each of the four conditions was completed once in a random order in each block of trials. Three blocks were presented for each task. The mean threshold across the three blocks was taken as the final mean for each condition. Where geometric tracking was used, a geometric average of the means was calculated. 10-ms ramps were used to gate the onset and offset of all stimuli, unless otherwise specified, and these are included in all stimulus durations reported.

For three of the psychophysical discrimination tasks a two-alternative forced-choice paradigm was used in which the listener was asked to detect which of two observation intervals, each consisting of four stimuli (AAAA vs ABAB), contained non-identical stimuli (Hopkins and Moore, 2010). This paradigm has been shown to minimize practice effects (e.g. King et al., 2013). For these tasks, there was a 50-ms silent period between stimuli within one of the two observation intervals, and a 500-ms silent period between observation intervals. Minimal training was given, with the experimenter confirming that the participant understood the task via a brief discussion after hearing the stimuli and by observing two correct responses on a brief practice run. The numbered intervals were visually cued with white lights on a computer screen for the duration of each stimulus and feedback was given in the form of a red or green light for incorrect and correct responses, respectively. Participants made their responses using numeric buttons on a computer keyboard. The participant could take a break between adaptive tracks and did not commence another test sequence until they indicated they were ready. Specific details for each of the tasks performed are given in the following sections.

#### 2.4.1 Frequency difference limens (FDLs)

The AAAA vs. ABAB paradigm was used. Tones were 200 ms in duration. Stimulus levels of 40 and 80 dB SPL were used for each frequency. The low-frequency standard stimulus (A) was a 255-Hz pure tone. For the high-frequency condition, the stimulus was a transposed tone, consisting of a 4000-Hz carrier modulated by a half-wave rectified, and low-pass filtered, pure tone (using a fourth-order Butterworth filter with a cutoff frequency of 2040 Hz). Transposed tones were used as they are designed to produce equivalent neural temporal firing patterns in high-frequency spectral regions of the cochlea as occur in low-frequency spectral regions in response to a pure tone (Bernstein and Trahiotis, 2002). For the standard stimulus (A) the frequency of the pure tone modulator was 255 Hz. Note that for the high-frequency condition, the task was modulation frequency discrimination. In both cases, the frequency of the pure tone for the comparison stimulus (B) was higher than that of the standard, and was varied adaptively. The starting difference in frequency was 10% and the frequency of the comparison stimulus was varied geometrically with an initial step size of a factor of 2 and a subsequent step size of √2. For the high-frequency conditions, low-pass pink noise was added in order to mask combination tones. The cut-off frequency of the noise band was 2500 Hz and the spectrum level at 1000 Hz was 40 dB below the signal level. Based on estimates of distortion product level by Oxenham et al. (2009), the noise should have masked any combination tones below 2500 Hz.

#### 2.4.2 Intensity difference limens (IDLs)

The AAAA vs. ABAB paradigm was used. Tones were 200 ms in duration. Stimuli were pure tones presented at 255 or 4000 Hz, and at two levels (40 and 80 dB SPL for the standard, A, stimuli). The comparison stimulus (B) was higher in level than the standard. The starting Weber fraction was 10 dB and was varied arithmetically with an initial step size of 4 dB and a second step size of 2 dB.

#### 2.4.3 Interaural phase difference discrimination (IPD)

The AAAA vs. ABAB paradigm was used. Tones were 300 ms in duration (including 50 ms ramps). The low-frequency stimulus was a 255-Hz pure tone and the high-frequency stimulus was a transposed tone, consisting of a 4000-Hz tonal carrier modulated by a half-wave rectified, and low-pass filtered, 255-Hz pure tone. Stimulus levels of 40 and 80 dB SPL were used for each frequency. The interaural phase difference for the standerd stimulus (A) was always zero. For the comparison stimulus (B) the interaural phase difference for the pure tone or pure-tone modulator was varied adaptively. The starting difference was 30°, generated by advancing the phase for the right-ear signal. The interaural phase difference was varied geometrically using an initial step size of a factor of 1.56 and a second step size of 1.25. The maximum interaural phase difference for stimulus B was restricted to 90°. If the maximum difference was reached, the difference remained fixed until two correct responses were given consecutively. For the high-frequency conditions, low-pass pink noise was added in order to mask combination tones. The cut-off frequency of the noise band was 2500 Hz and the spectrum level at 1000 Hz was 40 dB below the signal level.

#### 2.4.4 Amplitude modulation detection (AMD)

A three-alternative forced-choice paradigm was used. Stimuli were 200 ms in duration. Carriers were 255-Hz and 4000-Hz pure tones for the low- and high-frequency stimuli respectively, and the target stimulus was a carrier sinusoidally amplitude modulated at 25 Hz. Carrier levels of 40 and 80 dB SPL were used for each frequency. The RMS energy was equated across intervals. The starting modulation depth was 50% and this was then geometrically varied according to a two-down one-up track with an initial step size factor of 1.56 and a final step size factor of 1.25. There was a 500-ms inter-stimulus interval between each of the three tones.

#### 2.4.5 Digit triplet test (DTT)

In the DTT, the participant is required to identify three spoken digits presented sequentially in a background noise (Smits et al., 2004). The digits were in the range 1–9 and the correct identification of all three was required for a correct response. The digits were voiced recordings from a single speaker taken from McShefferty et al. (2013). The noise was speech-shaped and fixed at each of two levels (40 and 80 dB SPL) while the sound level of the spoken digits was varied. A method of constant stimuli was used, with six repetitions at each of eight signal-to-noise ratios (SNRs). Each SNR/level combination was presented once, in a random order, in each block of trials. Three blocks were presented, and the overall percent correct responses calculated for each condition. The SNRs used were −24 to −3 in steps of 3 dB. The stimulus began with 200 ms of noise before the first digit was presented. There was a 50-ms interval between each of the spoken digits and the noise was continuous. Participants made their response by selecting three tick-boxes covering the range 1–9 using a computer mouse and then confirming their selection. Visual feedback was given in the form of a green (correct) or red (incorrect) light. For each individual, a cumulative Gaussian was fitted to the data to model the distribution and to allow the SNR to be estimated for a range of response rates. The results section uses 25%, 50% and 75% correct points to give an overview of the psychometric function.

#### 2.4.6 Co-ordinate response measure (CRM)

In the CRM speech task (Bolia et al., 2000), the participant is presented with a number of speech utterances of the structure “*Ready* < *call sign* > *go to* < *color* > < *number* > *now*”, in which there are eight unique callsigns, four different colors (Blue, Red, White, Green) and the number is in the range 1–4. The participant's callsign was always “Baron” and they were instructed to listen for the speaker who said “*Ready Baron*” and identify the color and number spoken by that speaker. The gender and identity of the target was changed on each trial and there were four male and four female speakers. Two maskers were presented simultaneously, which were always different speakers and different callsigns, although the color and number could match that of the target. All stimuli were spoken by native British-English speakers (Kitterick et al., 2010).

The CRM was performed at two sound levels (40 and 80 dB SPL) which defined the level of the combined masker stimuli, and in two different masker configurations; one where the maskers were presented centrally (CRMc) and one in which they were offset by 60° azimuth on either side of the mid-line (CRMo). This was achieved by multiplying the acoustic stimuli by head-related impulse responses from the CIPIC database (Algazi et al., 2002). In each trial the target sentence was presented centrally at a sound level which varied trial-by-trial and the maskers were presented at a fixed sound level. A method of constant stimuli was used, with six repetitions at each of eight SNRs. Each offset/level/SNR combination was presented once, in a random order, in each block of trials. Three blocks were presented, and the overall percent correct responses calculated for each condition. The SNRs used were −6 dB to +8 dB in steps of 2 dB for the central condition and −14 dB to 0 dB in steps of 2 dB for the offset condition. Participants indicated their response by clicking on one of 16 buttons on the computer display, arranged in four color-coded columns with each row identified as a separate number. Visual feedback was given in the form of a green (correct) or red (incorrect) light. For each individual, a cumulative Gaussian was fitted to the data to model the distribution and to allow the SNR to be interpolated for a range of response rates. The results section uses 25%, 50% and 75% correct points to give an overview of the psychometric function.

#### 2.4.7 Localization task (LOC)

The auditory localization task was performed for levels of 40 and 80 dB SPL. A single spoken word (“Tiger” taken from the CRM corpus) was heard in quiet presented over headphones after being multiplied by one of 17 head-related impulse responses (Algazi et al., 2002), intended to make the percept originate from one of the following virtual azimuths: ±80, 65, 55, 45, 35, 25, 15, 5 and 0°. Each speech token was presented with zero degrees elevation. Participants indicated their response by clicking one of 17 boxes on the computer display laid out schematically in a semi-circle, as if looking down on the participant perceiving the sound source. Each location was presented six times in a single run, and three runs were completed for each sound level. Both the order of the runs and of the stimuli within a run were randomized. No feedback was provided.

#### 2.4.8 Musical consonance task (CON)

There is evidence that ratings of the perceived pleasantness of chords are related to the strength of neural temporal coding (Bones et al., 2014), and temporal coding has been linked to synaptopathy (Bharadwaj et al., 2014). Hence a consonance preference task may be effective at identifying temporal coding deficits due to synaptopathy. The stimuli and methodology were based on Bones and Plack (2015). Two-note chords (dyads) were created by combining each of eight complex tones (fundamental frequencies, F0s, of 293.66, 311.12, 329.62, 349.22, 370, 392, 415.3, 440 Hz) with each of 11 higher-F0 tones. Each complex tone contained 20 equal-amplitude harmonics. Each dyad is named after the musical interval between the F0s of the high and low notes (ratios of 1.06, 1.12, 1.19, 1.26, 1.33, 1.41, 1.50, 1.59, 1.68, 1.78, 1.89). Dyads were 2 s in duration, including 10 ms raised-cosine onset and offset ramps. Each dyad was preceded by Gaussian noise with the same duration and filtering (low-pass filtered at 6000 Hz). A 500-ms silence separated the noise and the dyad. The purpose of the noise was to prevent trials being influenced by the preceding stimulus and thus biasing the pleasantness judgement of the current trial (McDermott et al., 2010). Listeners were asked to rate how pleasant or unpleasant they found the chord using a seven-point Likert scale (−3 to +3). The harmonics of each note had the same amplitude, and the overall level of each dyad was 80 dB SPL.

#### 2.4.9 Self-report assessment of hearing ability

The SSQ was used to allow listeners to report their hearing ability in several domains, which are split into three scales; speech, spatial and qualities of hearing. The questionnaire consists of 49 questions which describe a listening situation and ask people to rate their listening ability in that situation from 0 to 10, with a higher number indicating better performance and an improved sense of hearing ability. The SSQ is designed to provide a comprehensive assessment of an individual's perceived ability to hear in the real world. The Speech scale (consisting of 14 items) covers an extensive range of realistic speech contexts that vary in their assumed difficulty. The items cover conditions of competing sound, the number of speakers, and selective attention (attending to one speech stream in a background of many), in an attempt to identify specific listening environments in which the ability to hear speech may be affected. The Spatial hearing scale (consisting of 17 items) addresses direction, distance, and movement discrimination abilities. The Qualities scale (consisting of 18 items) addresses issues related to the ability to segregate sounds, the clarity of sounds, and the demand of listening effort. For each individual, the mean score across all of the items on a scale was taken, which allows all three scales to be plotted on the same axis. This average score for each scale is used as a summary metric for each listener and can be compared to that listener's noise exposure score to ascertain if there is a relation between the two. A negative relation is predicted, with increasing noise exposure expected to be associated with a decreasing score, which would indicate a decrease in the listener's perceived hearing ability.

#### 2.4.10 Musical experience

To estimate the degree of musical experience, we asked those participants who reported having learnt an instrument: “Between what ages did you regularly play?” The total number of years of playing a musical instrument was taken as the metric of musical experience. A subset of participants worked in the music industry as sound engineers/technicians and these participants scored highly on this metric.

## 3 Results

Many of the behavioral thresholds were found to be non-normally distributed and so in these cases Spearman's rho was used in order to evaluate the extent to which lifetime noise exposure predicted performance. Due to attrition, the number of participants varies slightly for each task and so the number of participants included is noted for each task. Note that the primary focus here is on the relation of behavioral performance to noise exposure, not on the relations between behavioral measures. Due to the exploratory nature of this study, a large number of comparisons are performed. This approach comes with a multiple comparisons penalty, to the extent that potentially genuine, albeit weak, relations may be discarded. Therefore, no correction for multiple comparisons has been performed and discussion of the results considers any relation which reaches an alpha of 0.05, albeit with appropriate caveats.

### 3.1 Noise exposures

**Fig. 1:**
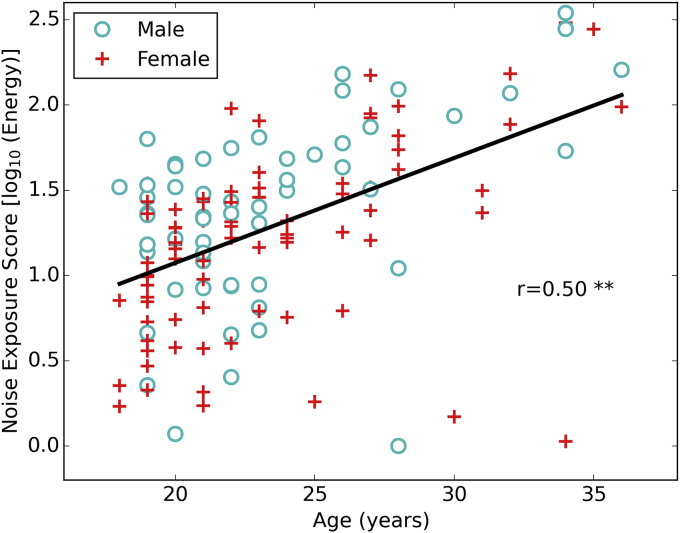
Noise exposure scores as a function of age for 137 participants. The regression line is plotted with the Pearson correlation coefficient shown in the text (* = 0.05, ** = p < 0.01).

Estimated lifetime noise exposure scores varied with respect to log(energy) from 0 to 2.54. In terms of energy, there was a difference of a factor of 300 between the lowest and the highest noise exposed participants. There was no significant difference between noise exposure scores for males (mean = 1.37, s.d. = 0.54) and females (mean = 1.22, s.d. = 0.51): t(136) = 1.63, p = 0.10. Therefore, the remaining results for male and female listeners were pooled. Noise exposure was used as the primary predictor variable in the analyses. Fig. 1 shows the distribution of noise exposures for the cohort as a function of age.

In addition to considering the entire cohort in correlational analyses, it is instructive to examine groups with extreme low and high noise exposure. This division into sub-groups may increase the likelihood of observing the effects of synaptopathy, and provides a concise and clear visual indication of the sensitivity of each measure to noise exposure. Hence, in the figures that follow, we present data for the 25% of the cohort (up to 34 individuals in each group, depending on the condition) with the lowest (green open squares) and highest (black filled squares) noise exposure scores. These groups had mean exposures (expressed on a logarithmic scale) of 0.63 (range; 0–0.95) and 1.95 (range; 1.60–2.54), respectively. Across the different tasks, the number of individuals included sometimes changed slightly due to attrition, though the mean exposures were always close to those presented here.

### 3.2 Audiometric data and tinnitus

**Fig. 2:**
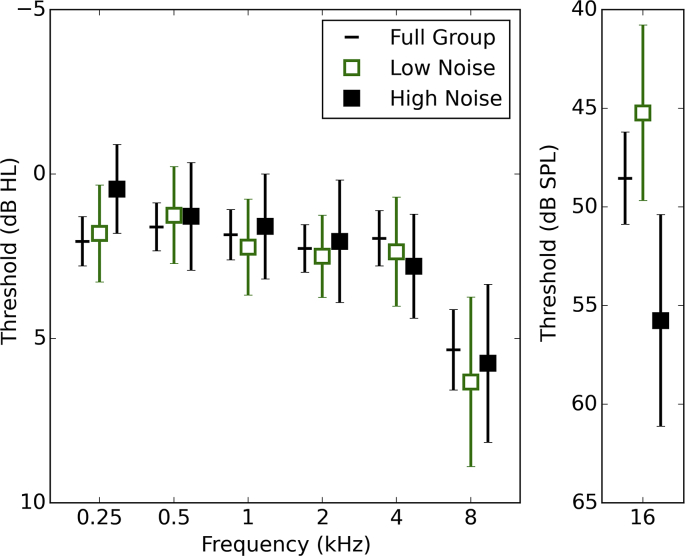
Pure tone audiometric thresholds (averaged across ears and listeners) are shown, with 95% confidence intervals, for the whole group and for the 25% of participants with the highest and lowest noise exposures.

Fig. 2 shows audiometric data (averaged across the ears) for all listeners, and for the low- and high-exposure groups. There was very little effect of noise exposure on audiometric threshold for frequencies up to 8 kHz, although there was a substantial difference between groups at 16 kHz, with the high-exposure group having poorer hearing thresholds on average. The Pearson correlation between 16-kHz audiometric thresholds and lifetime noise exposure was statistically significant (r = 0.29; p < 0.001), as also reported by Prendergast et al. (2017) using a near-identical dataset. Pearson correlation coefficients revealed no significant relation between audiometric threshold and noise exposure at 2000 Hz (r = 0.09; p = 0.27), 4000 Hz (r = 0.10; p = 0.24), and 8 kHz (r = 0.02; p = 0.82). Musical experience showed no statistically significant relation with any of the audiometric thresholds tested.

Ten participants reported experiencing prolonged tinnitus. Three of these participants were in the lowest 25% of noise exposures and five in the highest 25%.

### 3.3 Psychophysics (FDL, IDL, IPD, AMD)

**Fig. 3:**
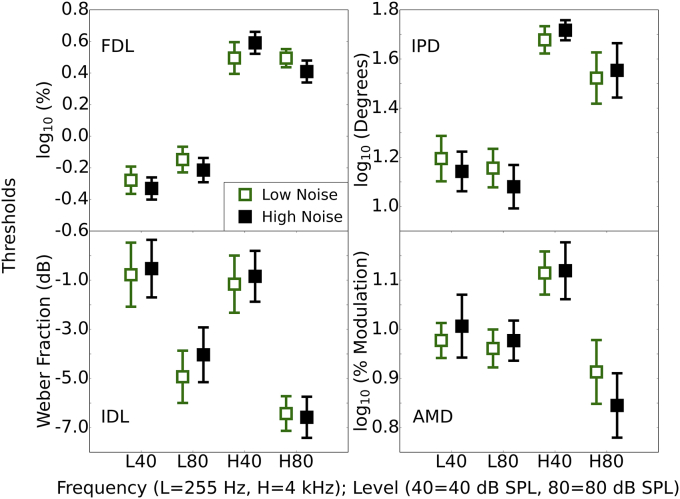
The four panels show the results of the four psychophysical tasks: Frequency difference limens (FDL), intensity difference limens (IDL), interaural phase difference discrimination (IPD), and amplitude modulation detection (AMD). Mean thresholds and 95% confidence intervals are plotted for the 25% of participants with the lowest and highest lifetime noise exposures for the four conditions of each task.

Fig. 3 shows the results for the four psychophysical experiments: FDL, IDL, IPD and AMD. In each panel, results for all four conditions are shown for each of the two levels and frequencies (L40, L80, H40, and H80, where “L” and “H” refer to low and high frequency, and the number refers to the level in dB SPL). There were no marked differences between the two groups, and small confidence intervals, which suggest that any effects of exposure were small.

Table 1 shows Spearman correlations for the whole group (with the N for each task indicated) between the noise exposure scores and the thresholds for each of the conditions. Table 1 also shows the correlation of noise exposure with two differential measures; one which contrasts different sound levels in the same spectral region (4000 Hz) and one which contrasts different spectral regions at the same sound level (80 dB SPL). It was predicted that cochlear synaptopathy would be associated with a positive correlation (increasing threshold with increasing noise exposure) in each case, based on the assumption that a positive correlation in the differential measure is caused by an elevation of threshold for high-noise exposed listeners in the H80 condition and equivalence of thresholds across exposure for the lower frequency/level condition. This assumption is premised on the low-SR fibers being primarily affected by noise exposure. There were only weak correlations, and these must be considered with caution as no correction for multiple comparisons was applied, and none of the significant correlations would survive Bonferroni correction.

**Table 1:** Spearman's rho coefficients are shown for the relation between thresholds for each of the four psychophysical tasks (and the two differential measures) and lifetime noise exposure, lifetime noise exposure controlling for musical experience, and musical experience. Conditions are labelled with the letter denoting frequency [(L)ow or (H)igh] and the numeric value indicating sound level (40 or 80, respectively). Positive correlations indicate results in the predicted direction (worse performance with increasing noise exposure) for the correlation and partial correlation with noise exposure. For musical experience, negative correlations indicate results in the predicted direction (better performance with increasing musical experience). * = p < 0.05; ** = p < 0.01 (uncorrected).

Task (N)	Condition					
	L40	L80	H40	H80	H80 – L80	H80 – H40
Correlation with noise exposure						
FDL (138)	−0.11	−0.09	0.14	−0.13	0.03	−0.23**
IPD (138)	−0.05	−0.16	0.03	0.08	0.19*	0.04
IDL (134)	−0.02	0.11	0.02	−0.06	−0.17*	−0.09
AMD (133)	0.02	0.08	−0.03	−0.20*	−0.24**	−0.21*
Correlation with noise exposure, controlling for musical experience						
FDL (138)	−0.01	0.15	0.06	−0.04	−0.03	−0.19*
IPD (138)	−0.04	0.07	−0.10	0.10	0.17*	0.04
IDL (134)	0.01	0.09	0.16	−0.03	−0.16	−0.09
AMD (133)	−0.11	0.04	0.16	−0.11	−0.21*	−0.18*
Correlation with musical experience						
FDL (138)	−0.26**	−0.36**	0.00	−0.22**	0.16	−0.16
IPD (138)	−0.05	−0.16	−0.08	−0.03	0.10	−0.00
IDL (134)	−0.08	−0.10	−0.19*	−0.23**	−0.07	−0.01
AMD (133)	−0.23**	−0.19*	−0.19*	−0.26**	−0.13	−0.12

The strongest relations across all tasks were in the opposite direction to those predicted. For the FDL task, increasing noise exposure was related to a reduction in the H80-H40 differential measure. This appears to be driven by high-noise participants outperforming low-noise participants in the high-frequency, high-level (H80), condition but performing more poorly in the high-frequency, low-level (H40) condition. For the IPD task there was a weak relation with noise exposure in the predicted direction with the differential measure, computed using H80-L80. This relation was partly driven by higher thresholds for the more exposed listeners in the H80 condition, as predicted, but it was driven more strongly by the noise-exposed listeners outperforming the less exposed in the L80 condition. There was also a weak effect for the IDL task, again for the differential measure computed using the two high-level conditions (H80-L80). This negative relation with noise exposure was driven by the fact that high- and low-noise exposed participants performed comparably in the H80 condition, but there was a decrease in performance with increasing noise exposure for the L80 condition.

The strongest relation of interest in the tasks presented is that between lifetime noise exposure and AMD, though it was in the opposite direction to that predicted. There was a negative relation between lifetime noise exposure and AMD threshold in the H80 condition; i.e. performance improved as noise exposure increased. The use of the differential frequency measure strengthened the relation, as in the L80 condition there was a slight decrease in performance with increasing noise exposure. The relations need to be validated in different cohorts to establish if they are in fact genuine, weak effects related to lifetime noise exposure.

Audiometric thresholds at 4000 Hz were found to correlate significantly with performance on the AMD high-frequency conditions, with high thresholds associated with better performance (H40, rho = −0.25; H80, rho = −0.31; H80 -H40, rho = −0.37; all p < 0.01). However, the correlations with noise exposure for these conditions showed a similar strength to the original correlations once audiometric sensitivity was controlled for (H40, rho = −0.25; H80, rho = −0.31; H80 -H40, rho = −0.37; all p < 0.01). No other behavioral measure (including those reported in sections 3.4, 3.5, 3.6) varied significantly as a function of 4000 Hz audiometric threshold.

### 3.4 Speech measures (DTT, CRM)

**Fig. 4:**
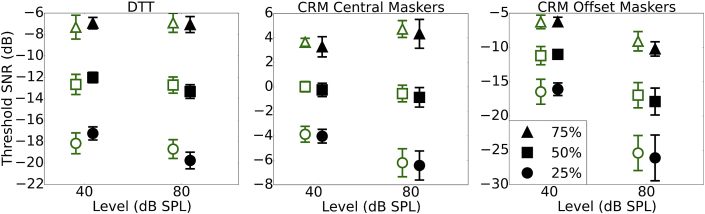
Mean thresholds (and 95% confidence intervals) are shown for the DTT, CRMc and CRMo speech tasks. The SNRs required for 25%, 50% and 75% correct on the psychometric function are plotted for the 25% of participants with the highest and lowest noise exposures in black (closed) and green (open) symbols, respectively. (For interpretation of the references to colour in this figure legend, the reader is referred to the web version of this article.)

Fig. 4 shows a summary of performance for each of the three speech tasks used; the DTT, the co-ordinate response measure with central maskers (CRMc), and the co-ordinate response measure with maskers spatially offset (CRMo). The SNRs at which 25%, 50% and 75% correct performance was estimated to occur are plotted for the low- and high-noise exposed groups. In each case, the differences between the groups are small.

Table 2 summarizes the relations between noise exposure and performance across the full group of participants (with N specified for each task). The relations were weak: None of the significant effects would survive correction for multiple comparisons and therefore must be interpreted with caution. The DTT showed a relation between performance and lifetime noise exposure for the differential measures taken at 25% and 50% correct on the psychometric function in the opposite direction to that predicted, i.e. with improving performance (a decrease in SNR) as a function of lifetime noise exposure. The effect was strongest at 25% correct, which was driven by highly noise-exposed listeners performing more poorly than lower noise-exposed listeners at 40 dB SPL and outperforming them at 80 dB SPL (a moderate but insignificant relation). A similar pattern was seen for 50% correct on the psychometric function, although it was weaker and only reached significance for the differential measure.

**Table 2:** Spearman's rho coefficients are shown for the relation between threshold on the speech tasks (and the differential measure) and lifetime noise exposure, lifetime noise exposure controlling for musical experience, and musical experience. Otherwise as Table 1.

Task (N)	Correlation with noise exposure			Correlation with noise exposure, controlling for musical experience			Correlation with musical experience		
	40 dB SPL	80 dB SPL	80–40 dB SPL	40 dB SPL	80 dB SPL	80–40 dB SPL	40 dB SPL	80 dB SPL	80–40 dB SPL
**DTT (139)**									
75%	0.12	−0.08	−0.11	0.10	−0.05	−0.08	0.08	−0.09	−0.10
50%	0.16	−0.12	−0.21*	0.12	−0.09	−0.16	0.11	−0.09	−0.17*
25%	0.17*	−0.13	−0.24**	0.14	−0.05	−0.20*	0.10	−0.07	−0.14
**CRMc (136)**									
75%	−0.19*	−0.01	0.09	−0.14	0.03	0.07	−0.15	0.10	0.04
50%	−0.18*	0.02	0.21*	−0.11	0.03	0.19*	−0.20*	−0.03	0.10
25%	−0.15	−0.06	0.21*	−0.08	0.09	0.19*	−0.19*	−0.04	0.10
**CRMo (136)**									
75%	−0.06	−0.11	−0.09	−0.02	−0.08	−0.10	−0.10	−0.07	−0.01
50%	−0.11	−0.09	−0.12	−0.05	−0.09	0.15	−0.16	−0.02	0.05
25%	−0.11	−0.10	−0.09	−0.05	0.11	0.11	−0.15	0.03	0.03

The CRMc task, in which the maskers were presented from the same spatial location as the target, revealed some weak trends. All three points on the psychometric function showed a qualitatively similar trend, with high noise-exposed listeners outperforming the less noise exposed in the 40 dB SPL condition and the groups being largely comparable for the 80 dB SPL condition.

There were no significant relations between noise exposure and performance on the CRMo task, in which the maskers were spatially offset. The 25% and 50% values for the CRMo task were extrapolated downwards from the range of SNRs tested (0 to −14 dB) and this extrapolation likely contributed in part to the increased confidence intervals for these values.

### 3.5 Localization task (LOC)

**Fig. 5:**
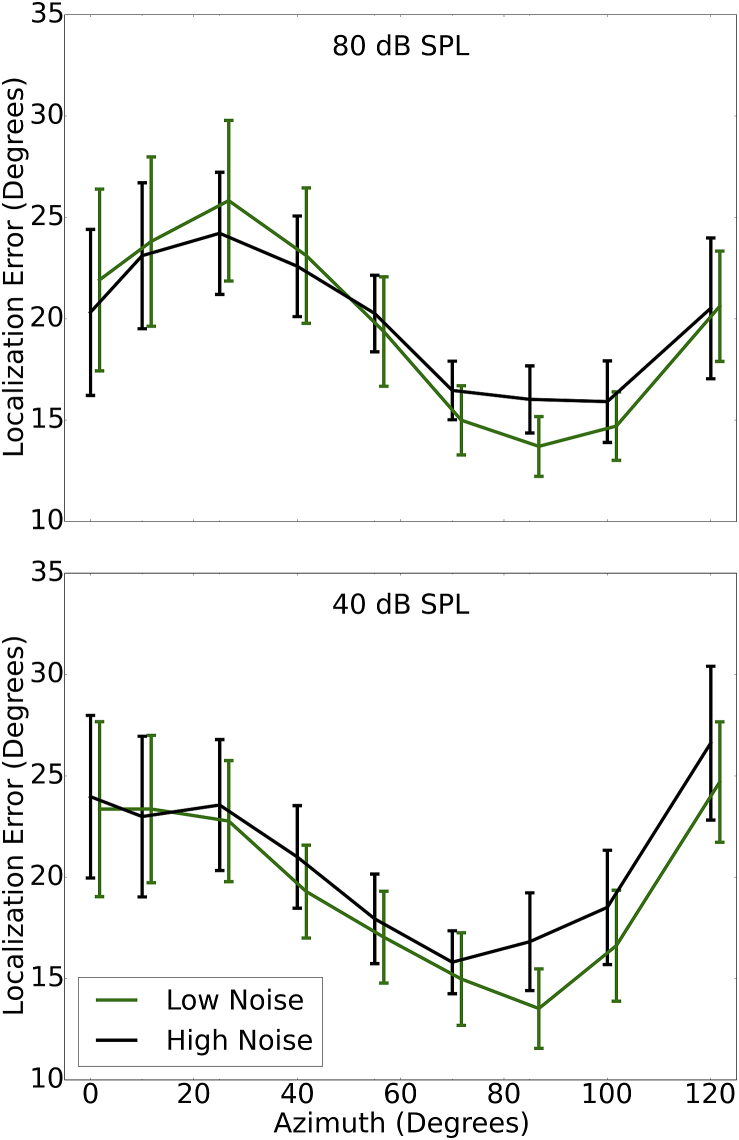
Mean localization error (and 95% confidence intervals) for the 25% of participants with the lowest and highest noise exposures (green and black lines, respectively). (For interpretation of the references to colour in this figure legend, the reader is referred to the web version of this article.)

Fig. 5 shows the average localization error in both conditions for the 25% of listeners with the lowest and highest levels of noise exposure (total N = 126; 31 participants in each of the two exposure groups). The results were averaged across the midline, such that each point is the average absolute error for both positive and negative azimuths. A summary error score was calculated for each participant by summing the mean absolute errors for each of the azimuths in order to correlate performance with noise exposure. Spearman's rho indicated no significant relation between noise exposure and localization error for either the 40 dB SPL (rho = 0.11, p > 0.05) or 80 dB SPL (rho = 0.04, p > 0.05) condition, nor was there a relation for the differential measure: the ratio between average errors at 80 and 40 dB SPL (rho = −0.02, p > 0.05).

### 3.6 Musical consonance (CON)

**Fig. 6:**
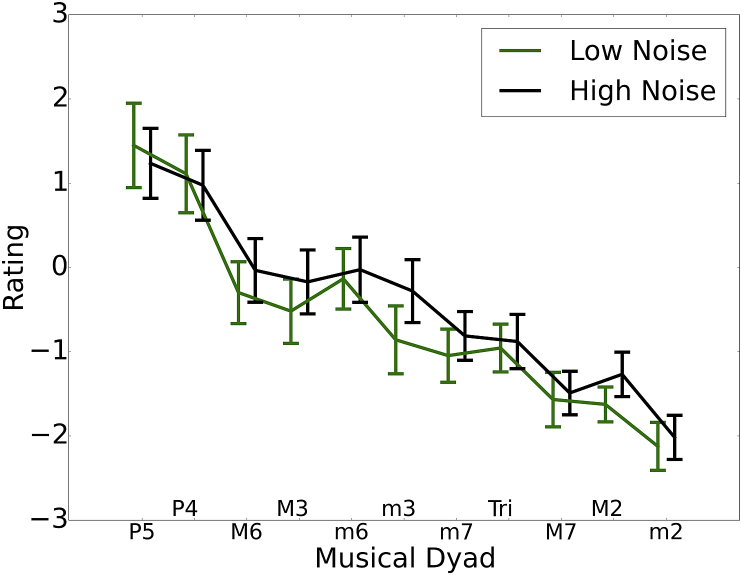
Mean pleasantness ratings are shown (along with 95% confidence intervals) for the 11 dyads in the consonance task. Results for the 25% of listeners with the lowest and highest lifetime noise exposures are plotted in green and black, respectively. (For interpretation of the references to colour in this figure legend, the reader is referred to the web version of this article.)

Fig. 6 shows the average rating for each of the 11 two-note chords for the 25% of highest and lowest noise-exposed participants (total N = 125, 31 participants in each of the exposure groups). Using a technique described by Bones and Plack (2015), a consonance preference score was calculated by taking the average z-score for the five most consonant chords and subtracting the average z-score for the five most dissonant chords for each participant. Spearman's rho indicated that this consonance preference score did not vary significantly with noise exposure (rho = 0.11, p > 0.05). The predicted direction was a reduction in consonance preference as a function of increasing lifetime noise exposure, due to a loss of temporal coding precision, which would mimic that observed in older listeners (Bones and Plack, 2015).

### 3.7 Speech, spatial, and qualities of hearing scale (SSQ)

**Fig. 7:**
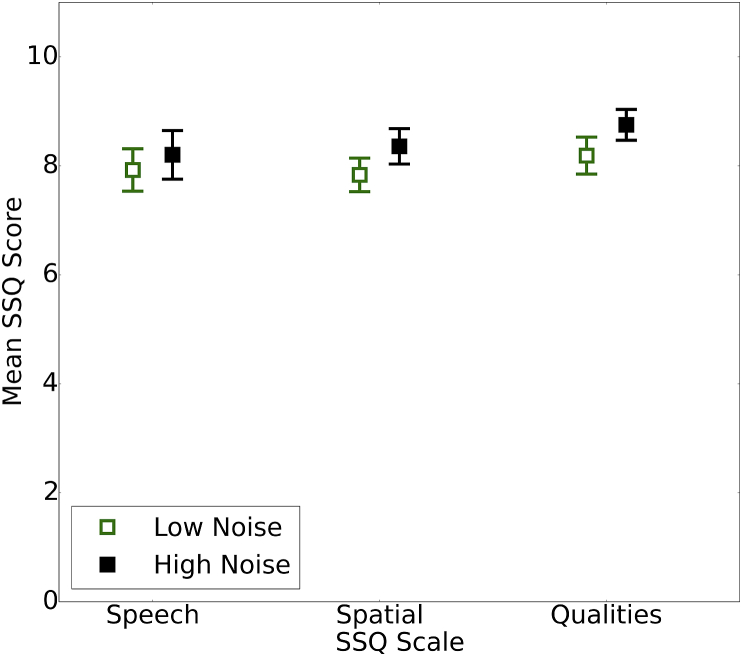
Mean ratings (and 95% confidence intervals) for the Speech, Spatial, and Qualities scales of the SSQ. Results for the 25% of listeners with the lowest and highest lifetime noise exposures are shown by green open squares and black solid squares, respectively. (For interpretation of the references to colour in this figure legend, the reader is referred to the web version of this article.)

Fig. 7 shows the average subjective rating on the three scales of the SSQ for the 25% of lowest and highest noise-exposed participants (total N = 135). A high SSQ score indicates good self-perceived hearing abilities. Contrary to the prediction, self-report hearing ability increased slightly with lifetime noise exposure for the Spatial (Spearmans rho = 0.17; p < 0.05) and Qualities scales (rho = 0.23; p < 0.01). No such relation was observed for the Speech scale of the questionnaire. The relation between lifetime noise exposure and SSQ score was statistically significant for two of the three scales. However, this was achieved by virtue of a large sample size and low variability across ratings. The mean difference between the groups was <1, which is the unit of granularity in the measure. Therefore, although these relations may indicate an underlying difference in perceived hearing ability that is of interest and potentially important to characterize, these differences are not of clinical relevance.

### 3.8 Relation of behavioral measures to musical experience

Musical experience correlated positively with noise exposure (rho = 0.38; p < 0.001). Hence musical experience could have been a confound, with the deleterious effects of noise exposure compensated by the performance benefits associated with musical experience (Parbery-Clark et al., 2009, Zendel and Alain, 2009, Yeend et al., 2017). Table 1 reports two further correlational analyses for the psychophysical measures: one in which partial correlations were performed between performance and lifetime noise exposure, with musical experience controlled. The second analysis is for the correlation of performance and musical experience.

Of the six correlations with noise exposure, all but one remained of a similar strength after controlling for musical experience. The relation between AMD H80 and noise exposure was markedly reduced, but four of the remaining five AMD conditions remained significant at the 0.05 level. The correlations indicate that AMD and FDL performance improved significantly with increased musical experience. However, none of the differential measures were significantly correlated with musical experience.

A similar pattern was seen for the speech tasks (Table 2), with the differential measures for the DTT and CRMc tasks having the strongest correlations with noise exposure once musical experience was controlled. The correlation with musical experience reached significance for the 40 dB SPL condition of the CRMc task (at 25% and 50% correct) but the differential measures did not. The differential measure for 50% correct on the DTT showed a significant correlation with musical experience.

Scores for the localization and musical consonance tasks showed no significant correlations either with musical experience controlled, or with musical experience on its own. For the SSQ, scores for the three scales were not significantly correlated with musical experience. However, the partial correlations between noise exposure and SSQ score, controlling for musical experience, were 0.12, 0.19 (both p < 0.05) and 0.23 (p < 0.01) for the Speech, Spatial and Qualities components, respectively. This suggests that the relation initially shown between subjective report of hearing ability and noise exposure is not related to the degree of musical experience reported by the listener.

### 3.9 Relation of behavioral measures to electrophysiological measures of synaptopathy

One core assumption of this study was that increased lifetime noise exposure is a proxy for increased levels of synaptopathy. Prendergast et al. (2017) used a largely identical dataset and found no relation between lifetime noise exposure and objective physiological measures of synaptopathy. One reason for this, which was discussed in that paper, is that supra-threshold ABR measures may not be sensitive enough to detect subtle changes in auditory processing. However, it could be argued that better estimates of synaptopathy can be obtained by using the electrophysiological measures, with the assumption that a weaker evoked response is indicative of greater underlying synaptopathy. To address this issue, we looked at how our battery of measures related to two core differential measures of synaptopathy, the wave I/V amplitude ratio and the FFR responses reported in Prendergast et al. (2017). The FFR (expressed in dB SNR) generated in response to a 255-Hz pure tone was used to assess the relation with low-frequency psychophysical conditions. The envelope FFR (expressed in dB SNR), generated in response to the modulated waveform (255 Hz modulation) of a 4000 Hz carrier was used to assess the relation with the high-frequency psychophysical conditions. The differential FFR measure was obtained by subtracting the SNR for the low-frequency FFR from the SNR for the envelope FFR, and this was used to assess any relation with differential behavioral measures. The differential FFR was also used to investigate whether there was any association with performance on the speech, musical consonance, localization, and SSQ measures.

The wave I/V ratio at 100 dB peSPL showed no significant relation with scores for any of the psychophysical, speech, musical consonance, or localization tasks, or the SSQ measures (p > 0.05 for all tests). The correlations of the FFR measures with behavioral performance are reported in Table 3. There were no significant relations between the differential FFR measure and the speech, musical consonance or localization tasks, nor the SSQ measure. The correlations with the psychophysical thresholds were generally weak. For all four psychophysical tasks, performance on the L40 condition showed a negative correlation with the FFR SNR in response to a 255-Hz pure tone. This association was strongest, and reached significance, for the AMD task. This AMD condition previously showed no relation with noise exposure and the effects of cochlear synaptopathy were expected to be observed in the high-frequency envelope FFR, rather than the low-frequency FFR. None of the differential behavioral measures showed a significant relation with the differential FFR measure.

**Table 3:** Spearman's rho coefficients are shown for the relation between the FFR measures and threshold for each of the behavioral tasks. Conditions are labelled with the letter denoting frequency [(L)ow or (H)igh] and the numeric value indicating sound level (40 or 80 dB SPL, respectively). For the L40 and L80 conditions, correlations were with the 255-Hz pure-tone FFR. For the H40 and H80 conditions, correlations were with the envelope FFR for a 4000-Hz carrier amplitude modulated at 255 Hz. For the differential measures (H80-L80, and H80-H40), correlations were with the differential FFR measure (envelope FFR minus pure-tone FFR). In each case, the predicted relation between the FFR measure and performance is a negative one. Those with noise-induced synaptopathy are expected to have lower FFR scores and poorer (higher) psychophysical thresholds. * = p < 0.05; ** = p < 0.01 (uncorrected).

Task (N)	Condition					
	L40	L80	H40	H80	H80 – L80	H80 – H40
FDL (123)	−0.08	0.00	−0.06	0.08	−0.03	0.09
IPD (123)	−0.12	0.05	0.04	−0.06	−0.15	−0.13
IDL (119)	−0.08	−0.15	−0.09	0.04	−0.01	0.00
AMD (119)	−0.19*	−0.06	0.04	0.15	0.12	0.11

### 3.10 Relation of behavioral measures to 16-kHz audiometric thresholds

Liberman et al. (2016) suggested that high-frequency audiometry may be a marker for cochlear synaptopathy at lower frequencies. To test this prediction, Spearman's rho correlations were computed between 16-kHz audiometric thresholds and scores for each of the behavioral tasks.

For the psychophysical tasks all the individual and differential measures were used. The 80 dB SPL condition was used for the speech, localization, and musical consonance tasks. The only task whose scores showed a significant relation with 16 kHz thresholds was AMD. In the H80 condition, performance improved with increasing 16-kHz thresholds (rho = −0.25; p < 0.01), although this relation was markedly reduced when a partial correlation was performed which controlled for the audiometric pure tone average at 2000 Hz, 4000 Hz and 8 kHz (rho = −0.15; p > 0.05). For the H80 – L80 differential measure, the relation was similar, performance improving with reduced 16-kHz audiometric sensitivity (rho = −0.26; p < 0.01) and for this condition the relation persisted after controlling for low-frequency audiometric thresholds (rho = −0.18; p < 0.05). These relations were in the opposite direction to that predicted on the basis of synaptopathy, as the expected effect of greater noise exposure (and therefore potentially greater synaptopathy) would be to reduce the fidelity of temporal coding and elevate behavioral thresholds. However, it is known that sensorineural hearing loss is often associated with improved AMD thresholds (e.g. Füllgrabe et al., 2003). From this perspective, relating to OHC dysfunction in participants with a high-frequency audiometric loss, the correlations were in the predicted direction. The H80 – H40 differential measure showed a comparable trend, with performance improving with increasing 16-kHz threshold (rho = −0.27; p < 0.01) and this trend persisted after correcting for low-frequency audiometric thresholds (rho = −0.23; p < 0.01). The H80, and both differential conditions, were the only conditions in which performance on the AMD task varied with high-frequency thresholds. The speech, localization, and musical consonance tasks, in addition to IPD, FDL, IDL, and SSQ, did not show any significant relation with 16-kHz thresholds (p > 0.05). The pure tone average of the 2000 Hz, 4000 Hz and 8 kHz audiometric thresholds was positively related to audiometric sensitivity at 16 kHz (r = 0.31; p < 0.01).

## 4 Discussion

The main aim of this study was to establish whether performance on a range of behavioral tasks varies as a function of lifetime noise exposure for young listeners with normal audiograms. Overall, there was no strong evidence that performance is affected by noise exposure. There were some weak trends which may be of interest for further study, but these did not survive correction for multiple comparisons. This study provides further evidence that any effects of cochlear synaptopathy are difficult to observe in young human listeners with normal audiograms.

### 4.1 Psychophysical results

The IDL, FDL and AMD thresholds are consistent with those in the literature for normal-hearing listeners (e.g. Viemeister and Bacon, 1988, He et al., 1998, Füllgrabe et al., 2003, Moore and Ernst, 2012). Whilst the IPD thresholds for the transposed stimuli are larger than those reported by Bernstein and Trahiotis (2002), they are comparable with IPD thresholds reported by Bharadwaj et al. (2015).

The basic psychophysical results indicate some weak relations of potential interest, although these are difficult to explain, as performance improved as a function of noise exposure for some conditions and declined for others. These contradictions were found both across conditions of the same task and across the different tasks. The strongest effects occurred for the differential measures, which attempt to account for some of the inherent variability across different listeners. Therefore, future investigations of sub-clinical hearing deficits, which are not readily identified from audiometric testing, may benefit from differential measures in order to reduce the impact of individual differences on performance (Plack et al., 2016). It must be noted however, that the differential measure based on level assumes that synaptopathy affects one condition (H80, in the context of the current study) and does not affect the lower-level condition (H40), which can then act as a within-subject control. Such an assumption is based on the evidence that low-SR fibers are primarily affected by noise exposure (Furman et al., 2013). If a specific fiber group is not targeted in this way in humans, then the results obtained with a differential measure based on level become more difficult to interpret.

The condition with the strongest relation with noise exposure was AMD for the high carrier frequency and high carrier level. However, this relation was counter to the predicted direction, as performance improved with increasing noise exposure. Similar effects have been reported in the literature when quantifying the modulation detection sensitivity of hearing-impaired listeners with a sensorineural hearing loss. Moore et al. (1996) reported that listeners with unilateral sensorineural hearing loss perceived enhanced envelope fluctuations in the impaired ear relative to the near-normal ear, possibly due to the loss of cochlear compression associated with OHC dysfunction. Also, Kale and Heinz (2010) reported enhanced envelope coding in auditory-nerve-fiber responses from noise-exposed chinchillas with permanent sensorineural hearing loss. Therefore, the relation between AMD performance and noise exposure may actually be driven by subtle differences in OHC function, an interpretation supported by the fact that 16-kHz thresholds were also related to AMD performance. Elevated thresholds at 16 kHz may be an early marker for sub-clinical OHC dysfunction in the standard audiometric range which is not detectable using pure-tone audiometry, although there was no effect of exposure on transient-evoked otoacoustic emission amplitudes, measured up to 4000 Hz, in the present cohort (Prendergast et al., 2017). Such an explanation would highlight the need to reconsider how we define “normal” hearing for the purposes of research studies and may have interesting implications for future investigations of sub-clinical processing deficits, but would contribute little to our understanding of noise-induced cochlear synaptopathy.

### 4.2 Speech measures and self-report

The DTT and the CRMc results both revealed weak relations with noise exposure that were again non-significant after correction. However, for the CRMc task, the relation with noise exposure was primarily observed for the 40 dB SPL condition, showing an improvement in performance with increasing noise exposure. This is opposite to the effect reported by Liberman et al. (2016) for their low-level speech task. The DTT showed different effects across the two sound levels, with increasing noise exposure relating to decreasing performance at 40 dB SPL and increasing performance at 80 dB SPL. The effects for the DTT occurred at 25% and 50% correct on the psychometric function. It is possible that the effects of synaptopathy are more apparent in difficult listening conditions, which would be concordant with Liberman et al. (2016), who used both time compression and reverberation to increase the difficulty of the task and exacerbate the differences between low and high noise exposed individuals. This notion is also supported by recent behavioral data collected in rats. Lobarinas et al. (2017a) found a reduction in the ability to detect a narrowband of noise presented in an ongoing background noise after exposure to intense (109 dB SPL) noise. This was associated with a supra-threshold decrease in wave I amplitude, consistent with a loss of cochlear synapses. However, the behavioral reduction in sensitivity was only observed for the most challenging condition tested (20 dB SNR).

### 4.3 Effects of musical experience

FDL and AMD thresholds were found to vary strongly with musical experience. However, this was only seen when looking at individual conditions, and none of the differential measures showed such a relation. The partial correlations, which controlled for musical experience, resulted in a weaker relation between noise exposure and performance for a number of the individual psychophysical tasks. However, the differential measures resulted in more robust correlations, as the partial correlations controlling for musical experience were comparable in magnitude to the initial correlation with noise exposure.

Performance on the speech tasks was not clearly related to musical experience, with only the 40 dB SPL CRM task with central maskers showing a clear relation between years of musical experience and performance. In a pattern similar to that seen for the psychophysical tasks, when controlling for musical experience in a partial correlation, the coefficients decreased in magnitude but the differential measures remained largely unaltered, and still showed a weak, but significant correlation. For the SSQ estimate of hearing ability, the correlations with noise exposure were unchanged or increased after musical experience was controlled.

To summarize, the data presented here are consistent with recent work by Yeend et al. (2017) in that a participant's degree of musical training is predictive of their performance on a number of psychophysical and speech-in-noise tasks. This adds further complexity to a series of parameters which are already difficult to delineate; those with high-degrees of noise exposure tend to be older, possibly have poorer high-frequency hearing, and are also more likely to have musical training which leads to enhanced performance on a number of auditory tasks. The data presented in the current manuscript highlight the value of using a differential measure of performance, as it is these measures which are largely unchanged after controlling for musical experience. Using a differential estimate of performance in an individual may control for musical experience and allow a more direct measure of the effects of noise exposure.

### 4.4 Relation of behavioral measures to electrophysiological measures

Prendergast et al. (2017) reported, in a dataset largely overlapping with the current cohort, no clear changes in ABR or FFR as a function of lifetime noise exposure. The estimate of lifetime noise exposure is sub-optimal, but does appear to accurately differentiate those with high levels of noise exposure from those with much lower exposure. We approached the current study with the hypothesis that noise-induced synaptopathy may be too subtle to detect using auditory evoked potentials, and that behavioral changes may be more readily observed. Therefore, we maintained the assumption that greater lifetime noise exposure is a legitimate proxy for an underlying loss of cochlear synapses. A counter-argument would be that electrophysiological measures of auditory function are a better proxy for underlying cochlear synaptopathy. Such an approach would posit that those with weaker ABRs and FFRs have sustained a loss of cochlear synapses which accounts for this altered response and thus they should also exhibit poorer behavioral performance. However, the wave I/V ratio was found not to be predictive of performance on any of the tasks used in this study. The strength of these correlations was generally weaker than those for performance versus noise exposure. The FFR was found to be weakly predictive of performance when single conditions were considered separately and this was for the low-frequency FFR and not the envelope FFR for the high frequency region. The differential FFR was not predictive for the differential behavioral conditions. Hence, using the electrophysiological metrics as a marker for synaptopathy did not provide any further insight into the relation of synaptopathy to behavioral measures.

It has been reported previously that the strength of auditory evoked potentials in an individual is predictive of performance on psychophysical tasks for normal-hearing listeners (e.g., Bones et al., 2014, Bharadwaj et al., 2015). Such a relation, with stronger evoked responses being concordant with better behavioral performance, is consistent with temporal coding precision being crucial for accurate auditory perception. However, these results were not replicated in the present study. Furthermore, if such measures are to be used to better understand noise-induced cochlear synaptopathy, they must in some way be linked to the noise exposure history of the individual. The present approach assumes a simple relation between noise exposure and behavioral thresholds. The interpretation is complicated if different listeners have different degrees of susceptibility to suffering physiological damage from acoustic trauma. It may also be the case that an acoustic event is more damaging depending on when in the lifetime it occurs. It is currently unknown whether such factors affect the manifestation of cochlear synaptopathy in humans.

### 4.5 Can noise-induced synaptopathy in humans with normal audiograms be disregarded?

The lack of an effect of noise exposure on behavioral performance is consistent with Prendergast et al. (2017) and Guest et al. (2017), who found no systematic changes in the ABR or the FFR as a function of noise exposure. The current study, using a wide range of behavioral measures, further supports the idea that the amount of cumulative lifetime exposure to high intensity sounds is not related to meaningful changes in auditory perception in young, audiometrically normal adults. However, it is possible that behavioral performance is relatively insensitive to synaptopathy. Oxenham (2016) applied a theoretical model based on signal detection theory to demonstrate that a 50% loss of synapses would lead to a decrease in d-prime on a typical psychophysical task by a factor of √2, which is close to the limits of test sensitivity, and well within the range of expected variability across audiometrically normal young adults. This analysis suggests that, even if substantial synaptopathy occurs, it may be difficult to measure its effects on perception. There are also some potential limitations in our methodology that should be considered. One possible limitation is that the stimuli for the four psychophysical tasks were narrowband, and hence for the high-level conditions, off-frequency listening (particularly on the high-frequency side of the excitation pattern) may have contributed to performance. This may have reduced the impact of low-SR fiber loss by recruiting unsaturated high-SR fibers. Another possibility, discussed by Prendergast et al. (2017), is that our retrospective self-report measure of noise exposure is too unreliable to distinguish individuals in terms of potential synaptopathy. However, as we argued previously, the differences in estimated exposure between the lowest and highest exposed were so great that it is unlikely that meaningful effects were washed out by imprecision in the estimates. In addition, essentially the same noise measure was significantly predictive of tinnitus in a recent study (Guest et al., 2017), even though the range of exposures and number of participants were smaller than in the present study. This suggests that the measure is sufficiently sensitive to distinguish between participants in terms of exposure.

Despite these caveats, our findings across the three studies from our laboratory to date are consistent with the hypothesis that noise-induced cochlear synaptopathy is insignificant in young humans with normal audiograms. In animal models, it is possible to titrate the noise exposure so as to deliver the maximum intensity possible without permanent threshold shift. The exposures encountered by humans are not so precise, and it may be that exposures sufficient to significantly reduce the number of cochlear synapses are also likely to lead to a loss of OHC function and an elevation of audiometric thresholds, particularly at high frequencies. Dobie and Humes (2017) discussed the difficulties involved in extrapolating the exposure levels used in the animal work to the human listener. They used historical evidence from human studies, in which very intense laboratory exposures were used, and the degree of temporary threshold shift as a proxy for damage to the auditory system. They argued that human listeners require much higher exposures than rodents to produce equivalent damage. The studies that have reported a decrease in wave-I ABR amplitude with noise exposure (Bramhall et al., 2017) and an increase in SP/AP ratio (Liberman et al., 2016) also reported audiometric differences between the groups. As discussed previously, there are differences in sensitivity in the 3000–6000 Hz range in the Bramhall et al. (2017) study. Liberman et al. (2016) reported significant differences in audiometric sensitivity between low and high exposure groups at 10 kHz and above, and a non-significant difference between the two groups at 8 kHz.

There are two competing hypotheses regarding the relation of audiometric loss to the differences in ABR waveforms between the exposure groups observed in some studies. The first is that high-frequency threshold elevations, and perhaps mild low-frequency (<8 kHz) threshold elevations, are markers for synaptopathy, and that the electrophysiological effects are a direct result of noise-induced synaptopathy. A second hypothesis is that the electrophysiological effects and the high-frequency audiometric deficits share the same cause: basal hair cell dysfunction, as opposed to cochlear synaptopathy at lower frequencies. As Liberman et al. (2016) suggest, the use of high-frequency masking noise to remove the contribution from basal regions when making ABR recordings may help to differentiate between these hypotheses.
